# Supplementary material for: Identification of photoperiod-induced specific miRNAs in the adrenal glands of Sunite sheep (Ovis aries)
Source: Front Vet Sci. 2022 Jul 22;9:888207. doi: 10.3389/fvets.2022.888207 (PMC9354845; doi:10.3389/fvets.2022.888207)
Supplement: Supplementary file 1 [file Data_Sheet_1.ZIP › Supplementary file/miRDeep2 file.zip!/miRDeep2 file/Know_Structure/SPLP42A1/oar-mir-199a.pdf]

miRBase precursor : oar-mir-199a
Total read count : 113539
oar-miR-199a-3p read count : 74418
remaining reads : 39121

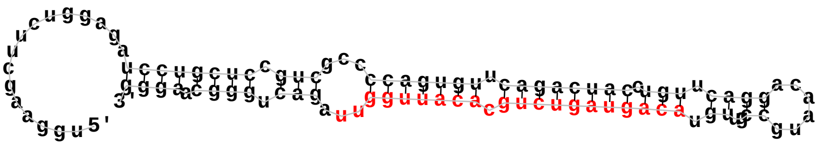

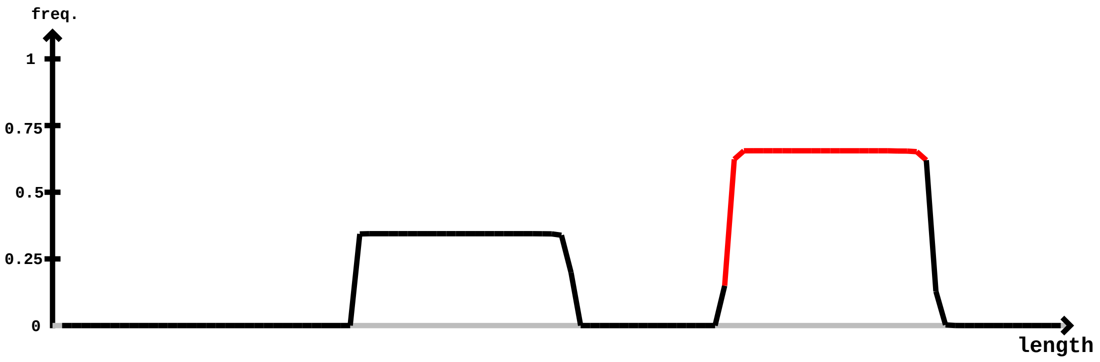

oar-miR-199a-3p

5'- uggaagcuucuggagauccugcuccgucgccccaguguucagacuaccuguucaggacaaugccguuguacaguagucugcacauugguuagacugggcaaggg -3' exp

................(((((((.(((...(((((((.((((((((.(((.((((......))..)).))))))))))).)))))))...))).)))).))).	reads	mm	sample
.............................ccccaguguucagacuaccugu...	1	0	seq
.............................ccccaguguucagacuaccuguu..	11	0	seq
.............................ccccaguguucagacuaccuguuc...	2	0	seq
..............................cccaguguucagacuaU...	1	1	seq
..............................cccaguguucagacuacc..	2	0	seq
..............................cccaguguucagacuaccu...	35	0	seq
..............................cccaguguucagacuaccug..	49	0	seq
..............................cccaguguucagacuaccugu...	572	0	seq
..............................cccaguguucagacuaccuguu..	15811	0	seq
..............................cccaguguucagacuaccuguuc...	22522	0	seq
..............................cccaguguucagacuaccuguucagg..	1	0	seq
...............................ccaguguucagacuaccu...	1	0	seq
...............................ccaguguucagacuaccuguu..	32	0	seq
...............................ccaguguucagacuaccuguuc...	70	0	seq
................................caguguucagacuaccuguu..	2	0	seq
................................caguguucagacuaccuguuc...	5	0	seq
.................................aguguucagacuaccuguu..	1	0	seq
.................................aguguucagacuaccuguuc...	1	0	seq
....aggacaaugccguuguacagua.............................	1	0	seq
....uguacaguagucugcacauuggu...............	1	0	seq
....guacaguagucugcacauugguu..............	1	0	seq
....uacaguagucugcacauu..................	25	0	seq
....uacaAuagucugcacauu..................	1	1	seq
....uacaguagucugcacauug.................	13	0	seq
....uacaguagucugcacauugg................	108	0	seq
....uacaguagucugcacauuggu...............	1814	0	seq
....uacaguagucugcacauugguu..............	13901	0	seq
....uacaguagucugcacauugguua.............	1102	0	seq
....uacaguagucugcacauugguuag............	10	0	seq
....acaguagucugcacauu..................	79	0	seq
....acaCuagucugcacauu..................	2	1	seq
....acaguagucugcacauug.................	10	0	seq
....acaguagucugcacauugg................	137	0	seq
....acaguagucugcacauuggu...............	1671	0	seq
....acaguagucugcacauugguu..............	39379	0	seq

oar-miR-199a-3p

```
uggaagcuucuggagauccugcuccgucgccccaguguucagacuaccuguucaggacaaugccguuguacaguagucugcacauugguuagacugggcaaggg
.....................................................................acaguagucugcacauugguua.............	12278	0	seq
.....................................................................acaguagucugcacauugguuag............	234	0	seq
.....................................................................acaguagucugcacauugguuaga...........	13	0	seq
.....................................................................acaguagucugcacauugguuagG...........	8	1	seq
......................................................................caguagucugcacauugg................	12	0	seq
......................................................................caguagucugcacauuggu...............	146	0	seq
......................................................................caguagucugcacauugguu..............	2563	0	seq
......................................................................caguagucugcacauugguua.............	853	0	seq
......................................................................caguagucugcacauugguuag............	37	0	seq
......................................................................caguagucugcacauugguuaga...........	5	0	seq
.......................................................................aguagucugcacauuggu...............	1	0	seq
.......................................................................aguagucugcacauugguu..............	11	0	seq
.......................................................................aguagucugcacauugguua.............	4	0	seq
```
